# Beyond the Central Nervous System: Uncovering Memantine’s Modulatory Role in the Peripheral Nervous System

**DOI:** 10.3390/medicines13030025

**Published:** 2026-08-21

**Authors:** Kyriaki Papadopoulou, Sophia Tsokkou, Ioannis Konstantinidis, Pavlos Pavlidis, Chrysanthi Sardeli, Dimitrios Kouvelas, Soultana Meditskou-Efthymiadou, Antonia Sioga, Theodora Papamitsou

**Affiliations:** 1 Laboratory of Histology-Embryology, Department of Medicine, Faculty of Health Sciences, Aristotle University of Thessaloniki, 54124 Thessaloniki, Greece; ikonstao@auth.gr (I.K.); sefthym@auth.gr (S.M.-E.); sioga@auth.gr (A.S.); thpapami@auth.gr (T.P.); 2 Department of Otorhinolarhingology, Head and Neck Surgery, University Medical Center Mainz, 55131 Mainz, Germany; papavlid@googlemail.com; 3 Department of Clinical Pharmacology, School of Medicine, Faculty of Health Sciences, Aristotle University of Thessaloniki, 54124 Thessaloniki, Greece; sardeli@auth.gr (C.S.); kouvelas@auth.gr (D.K.)

**Keywords:** memantine, NMDA receptor, peripheral nervous system, neuropathic pain, diabetic neuropathy, chemotherapy-induced peripheral neuropathy, phantom limb pain, retinal ganglion cell, neuroprotection, excitotoxicity

## Abstract

**Background:** Memantine, an uncompetitive and voltage-dependent N-methyl-D-aspartate (NMDA) receptor antagonist, is clinically established for moderate-to-severe Alzheimer’s disease. Its pharmacodynamic profile, low-to-moderate affinity, rapid open-channel block, and strong voltage dependency allows selective inhibition of pathological NMDA overactivation while preserving physiological neurotransmission. Increasing evidence shows that these same mechanistic principles operate in the peripheral nervous system, where NMDA receptors contribute to excitotoxicity, oxidative stress, neuroinflammation, and maladaptive nociceptive signaling. **Purpose:** To synthesize emerging preclinical and clinical evidence demonstrating memantine’s modulatory and neuroprotective actions in peripheral neurons and glia and to outline implications for drug repurposing across neurology, pain medicine, oncology, supportive care, and ophthalmology. **Methodology:** A narrative integration of mechanistic studies, in vivo preclinical models, and heterogeneous clinical trials evaluating memantine’s effects on peripheral sensory neurons, autonomic neurons, Schwann cells, retinal ganglion cells, and neuromuscular junction physiology. Evidence was examined across conditions involving excitotoxicity, oxidative injury, mitochondrial dysfunction, apoptotic signaling, neuroinflammation, and neuropathic pain amplification. **Results**: Memantine consistently attenuates peripheral excitotoxic calcium influx, suppresses NOX-2–mediated ROS generation, stabilizes mitochondrial membrane potential, modulates Bax/Bcl-2 signaling, and reduces neuroinflammatory cytokine activity. It also inhibits dorsal horn wind-up selectively under neuropathic conditions. These convergent mechanisms yield protective effects across chemotherapy-induced peripheral neuropathy (CIPN), diabetic neuropathy, traumatic nerve injury, phantom limb pain, retinal ganglion cell excitotoxicity, and organophosphate-induced neuromuscular toxicity. Clinical evidence includes improved multimodal neuropathy outcomes in diabetic neuropathy when combined with gabapentin, reduced phantom limb pain prevalence and intensity at six months, and a five-fold reduction in post-mastectomy neuropathic pain with pre-emptive administration. **Conclusions:** Memantine should be conceptually reframed as a system-wide neuroprotective agent with substantial translational potential beyond the CNS. Priorities for future development include NR2B-selective peripheral NMDA antagonists, peripherally restricted formulations, single-cell transcriptomic mapping of peripheral NMDA receptor subtypes, and adequately powered PNS-specific randomized trials.

## 1. Introduction

### 1.1. Historical Development and Clinical Trajectory

Memantine was first synthesized as a derivative of amantadine in the late 1960s and entered clinical use in the Federal Republic of Germany initially for indications including Parkinson’s disease and spasticity [1]. Its principal mechanism of action—uncompetitive, voltage-dependent blockade of the N-methyl-D-aspartate (NMDA) receptor ion channel—was characterized progressively from the 1970s through the 1990s by the seminal contributions of Lipton, Parsons, and colleagues, culminating in the recognition that its kinetic profile differed fundamentally from earlier NMDA antagonists such as phencyclidine and MK-801 [1,2]. Unlike these high-affinity agents, which produce dissociative and psychotomimetic adverse effects through near-irreversible channel blockade, memantine exits the open channel sufficiently rapidly during transient physiological excitatory postsynaptic potentials (EPSPs), thereby preserving the spatiotemporal specificity of synaptic transmission whilst blocking sustained, tonically elevated glutamate receptor activation, characteristic of neurodegenerative and neuropathic disease states [1,3].

The European Medicines Agency (EMA) granted the first centralized marketing authorization for memantine (Ebixa^®^, Lundbeck, Copenhagen, Denmark) on 15 May 2002 for the treatment of moderate-to-severe Alzheimer’s disease, with the indication subsequently extended by the European Commission in November 2005 to include moderate Alzheimer’s disease. United States Food and Drug Administration (FDA) approval for moderate-to-severe Alzheimer’s disease followed on 16 October 2003, based principally on two pivotal double-blind RCTs demonstrating superiority over the placebo on the Severe Impairment Battery (SIB) and Clinician’s Interview-Based Impression of Change (CIBIC-plus) instruments [4]. Memantine is also indicated for vascular dementia, Parkinson’s disease, and spasticity in various jurisdictions [5]. The CNS framing of these approvals consolidated a clinical and research culture that viewed memantine essentially as a cortical–hippocampal neuroprotective agent, an identity that, whilst accurate, was incomplete.

### 1.2. The Gap in Peripheral Neuroscience

The preponderance of memantine research has concentrated on the CNS, even though functional NMDA receptors were identified on peripheral sensory neurons, autonomic ganglia, Schwann cells, and non-neuronal cells of the peripheral nervous system decades ago [6,7]. In conditions ranging from painful diabetic neuropathy to CIPN, from phantom limb pain to traumatic nerve injury, peripheral NMDA receptor upregulation and glutamate excitotoxicity contribute substantially to the initiation, amplification, and maintenance of peripheral and central sensitization [8,9]. Memantine reaches tissue concentrations at peripheral nerve sites that are pharmacologically relevant to NMDA receptor blockade, yet its peripheral mechanisms have been systematically understudied relative to its central ones [5,10].

This review addresses that gap comprehensively. It synthesizes evidence across molecular pharmacology, cellular neuroscience, animal neuropathy models, and clinical RCTs to characterize memantine’s peripheral neuroprotective actions, delineate the conditions in which they are most clinically meaningful, and identify the translational and pharmacological priorities required to advance this underexplored dimension of memantine biology.

### 1.3. Aim, Scope, and Structure

This review is structured to address the following: (i) the pharmacology of memantine with respect to peripheral tissue distribution and non-NMDA receptor interactions; (ii) the distribution, functional roles, and subunit composition of peripheral NMDA receptors; (iii) the principal cellular mechanisms through which memantine protects peripheral neurons; (iv) evidence in specific peripheral neuropathy types; (v) memantine’s effects at the neuromuscular junction and autonomic ganglia; (vi) peripheral organ systems influenced by memantine; (vii) optic nerve and retinal pathology; (viii) clinical evidence synthesis across neuropathic pain syndromes; (ix) limitations of the current evidence base; and (x) future research directions, including novel delivery systems and clinical trial design. Throughout, mechanistic and clinical evidence are integrated to enable a unified assessment of the therapeutic potential of peripheral memantine application. To respect the functional anatomy of the peripheral nervous system, the clinical evidence in Section 5, Section 6 and Section 7 is organized around its two principal divisions. The somatic nervous system (voluntary motor efferents and sensory afferents) is covered in Section 5 (peripheral neuropathies of sensory afferents, including chemotherapy-induced peripheral neuropathy, diabetic peripheral neuropathy, small-fier and inflammatory neuropathies, phantom limb pain, and complex regional pain syndrome) and Section 6.1 (motor efferent terminal at the neuromuscular junction). The autonomic nervous system is covered in Section 6.2 (sympathetic and parasympathetic ganglia) and Section 7.2 (enteric nervous system). Downstream end-organ consequences of autonomic and vascular NMDA receptor signaling in the cardiovascular, gastrointestinal, and renal compartments are integrated in Section 7, and the retinal ganglion cell–optic nerve unit—a peripheral cranial-nerve extension—is treated separately in Section 8.

## 2. Pharmacology of Memantine Relevant to Peripheral Tissues

### 2.1. NMDA Receptor Blockade: Mechanism, Kinetics, and Voltage Dependence

**Figure 1:**
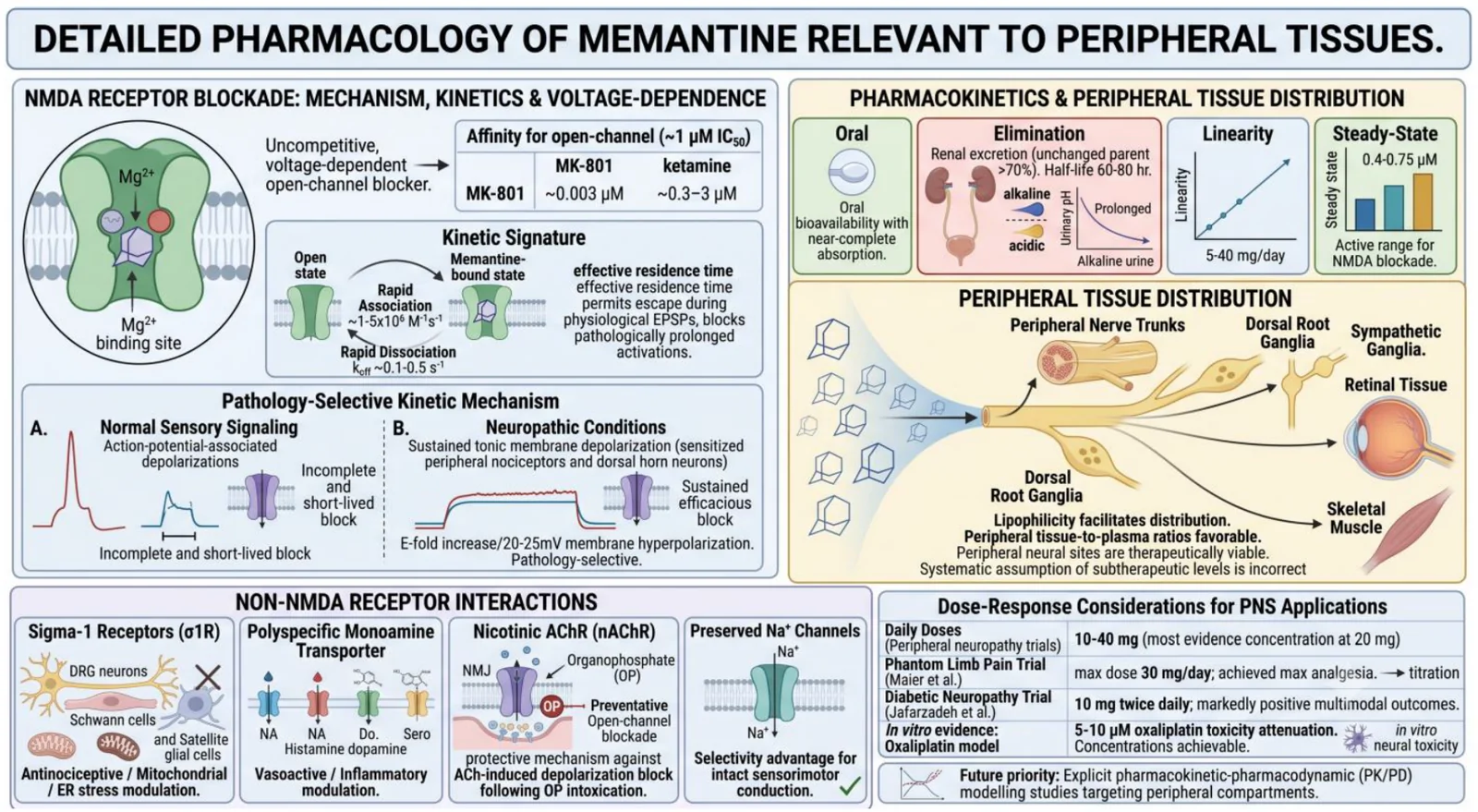
Detailed pharmacology of memantine relevant to peripheral tissues. Memantine is an uncompetitive, voltage-dependent, open-channel NMDA receptor blocker of moderate affinity (~1 μM IC_50_), whose rapid association and dissociation kinetics confer pathology-selective blocking—incomplete during normal sensory signaling but sustained under the tonic depolarization of neuropathic states. With near-complete oral absorption, predominantly renal elimination (half-life 60–80 h), linear kinetics, and steady-state levels (0.4–0.75 μM) within the active range, favorable lipophilicity drives distribution to peripheral nerve trunks, dorsal root and sympathetic ganglia, retina, and skeletal muscle, challenging the assumption of subtherapeutic peripheral exposure. Memantine also modulates sigma-1 receptors, monoamine transporters, and nicotinic acetylcholine receptors while sparing Na^+^ channels, supporting analgesic efficacy across peripheral neuropathy, phantom limb pain, diabetic neuropathy, and oxaliplatin neurotoxicity, with PK/PD modeling of peripheral compartments identified as a future priority.

Memantine occupies the Mg^2+^ binding site within the open NMDA receptor channel pore, acting as an uncompetitive, voltage-dependent, open-channel blocker. Its affinity for the open-channel state (~1 μM IC_50_) is substantially lower than that of MK-801 (~0.003 μM) or ketamine (~0.3–3 μM), and this reduced affinity is intrinsically coupled to its therapeutic tolerability [1,2]. The critical pharmacodynamic property that distinguishes memantine is not its affinity per se but its kinetic signature: rapid association with the channel pore upon opening (kon ~1–5 × 10^6^ M^−1^s^−1^) and comparably rapid dissociation upon membrane repolarization (koff ~0.1–0.5 s^−1^), resulting in an effective residence time in the nanomolar-to-low-micromolar range that is sufficient for blocking pathologically prolonged channel activations whilst permitting escape during physiological EPSPs [2,11] (Figure 1).

The voltage dependence of memantine block is steep, with the blocking efficacy increasing approximately e-fold per 20–25 mV of membrane hyperpolarization. This property means that, under tonic membrane depolarization characteristic of chronically sensitized peripheral nociceptors and dorsal horn neurons in neuropathic conditions, memantine block is both sustained and highly efficacious. Conversely, during the transient, action-potential-associated depolarizations of normal sensory signaling, the brief open time of the channel and rapid repolarization ensure that blocking is incomplete and short-lived [1,3]. This ‘pathology-selective’ kinetic mechanism is the pharmacological foundation for memantine’s ability to attenuate neuropathic sensitization without suppressing normal somatosensory function—a crucial property for any peripheral analgesic agent (Figure 1).

### 2.2. Interactions with Non-NMDA Receptor Systems

Clinically relevant concentrations of memantine interact with several receptor systems beyond the NMDA receptor. Memantine inhibits a poly-specific monoamine transporter capable of transporting noradrenaline, histamine, dopamine, and serotonin, and this pharmacological activity may contribute to vasoactive and inflammatory modulatory effects in peripheral tissues [10]. At higher concentrations, memantine demonstrates affinity for sigma-1 receptors (σ1Rs), which are expressed on DRG neurons, Schwann cells, and satellite glial cells, and which play significant roles in the modulation of neuropathic pain, mitochondrial function, and endoplasmic reticulum (ER) stress responses [12]. The σ1R interaction may be particularly relevant to memantine’s anti-hyperalgesia effects, as σ1R antagonism is independently anti-nociceptive in rodent neuropathy models.

Memantine also modulates nicotinic acetylcholine receptor (nAChR) ion channels, particularly the nicotinic receptor–sodium ionophore complex at the neuromuscular junction, through open-channel blockade [13]. This property, whilst potentially limiting in terms of therapeutic index for NMJ applications, provides a distinct protective mechanism against acetylcholine-induced depolarization block following organophosphate intoxication. Additionally, memantine does not affect the resting motor threshold or cortical silent period duration in transcranial magnetic stimulation (TMS) paradigms at clinical doses, indicating that voltage-gated sodium channel function is preserved—a selectivity advantage for peripheral analgesic applications requiring intact sensorimotor conduction [14] (Figure 1).

### 2.3. Pharmacokinetics and Peripheral Tissue Distribution

Memantine is orally bioavailable with near-complete absorption following oral administration. Its pharmacokinetics are linear across the clinical dose range (5–40 mg/day), with a prolonged elimination half-life of approximately 60–80 h in individuals with normal renal function, supporting once-daily or twice-daily dosing regimens [5]. The drug is primarily excreted by the kidney as an unchanged parent compound (>70%), with alkaline urine substantially prolonging elimination half-life through reduced tubular reabsorption. Steady-state plasma concentrations following 20–30 mg/day dosing are typically in the range of 70–130 ng/mL (approximately 0.4–0.75 μM), which is within the pharmacologically active range for NMDA receptor blockade in both central and peripheral compartments [14] (Figure 1).

Critically, memantine is highly lipophilic, a physicochemical property shared across adamantane derivatives, and this lipophilicity facilitates distribution into peripheral nerve trunks, dorsal root ganglia, sympathetic ganglia, retinal tissue, and skeletal muscle. While blood–nerve barrier penetration kinetics for memantine specifically have not been comprehensively characterized, the drug’s tissue-to-plasma ratios in peripheral neural compartments are likely to be at least as favorable as in the CNS, given that peripheral nerve barriers are generally less restrictive than the blood–brain barrier. However, in the absence of measured peripheral-nerve tissue-to-plasma partition coefficients (K_p_) or microdialysis data for memantine, this inference is a plausible working hypothesis rather than an established pharmacokinetic fact, and steady-state plasma concentrations (0.4–0.75 μM) sit at the lower end of the range required for robust NMDA receptor blockade in vitro. Direct tissue-level measurements are needed before subtherapeutic peripheral exposure can be either affirmed or excluded [5,10] (Figure 1).

### 2.4. Dose–Response Considerations for PNS Applications

Clinical trials targeting peripheral neuropathy have employed memantine at daily doses ranging from 10 mg to 40 mg, with most evidence concentrated at 20 mg/day (10 mg twice daily) [9,15]. The phantom limb pain RCT by Maier et al. titrated to 30 mg/day over four weeks, achieving maximal analgesic effects at this dose in the context of concurrent regional anesthesia [16]. In the diabetic neuropathy RCT by Jafarzadeh et al., memantine was titrated from 5 mg twice daily to 10 mg twice daily over four weeks, producing markedly positive multimodal outcomes [9]. In vitro studies in oxaliplatin-induced neuronal toxicity models employed memantine at 5–10 μM, an order of magnitude higher than steady-state human plasma concentrations of memantine at conventional doses (0.4–0.75 μM). Because dedicated peripheral-nerve tissue-to-plasma partition coefficient (K_p_) data for memantine are not available, the claim that these in vitro concentrations are matched at peripheral neural sites in patients is, at present, pharmacokinetically unverified, and should be treated as a working hypothesis pending direct tissue measurements [17]. The dose–response relationship for peripheral NMDA receptor blockade by memantine remains incompletely characterized relative to the CNS, and explicit pharmacokinetic–pharmacodynamic modeling studies targeting peripheral compartments are warranted (Figure 1).

## 3. NMDA Receptors in the Peripheral Nervous System

### 3.1. Expression in Dorsal Root Ganglia, Peripheral Axons, and Supporting Cells

**Figure 2:**
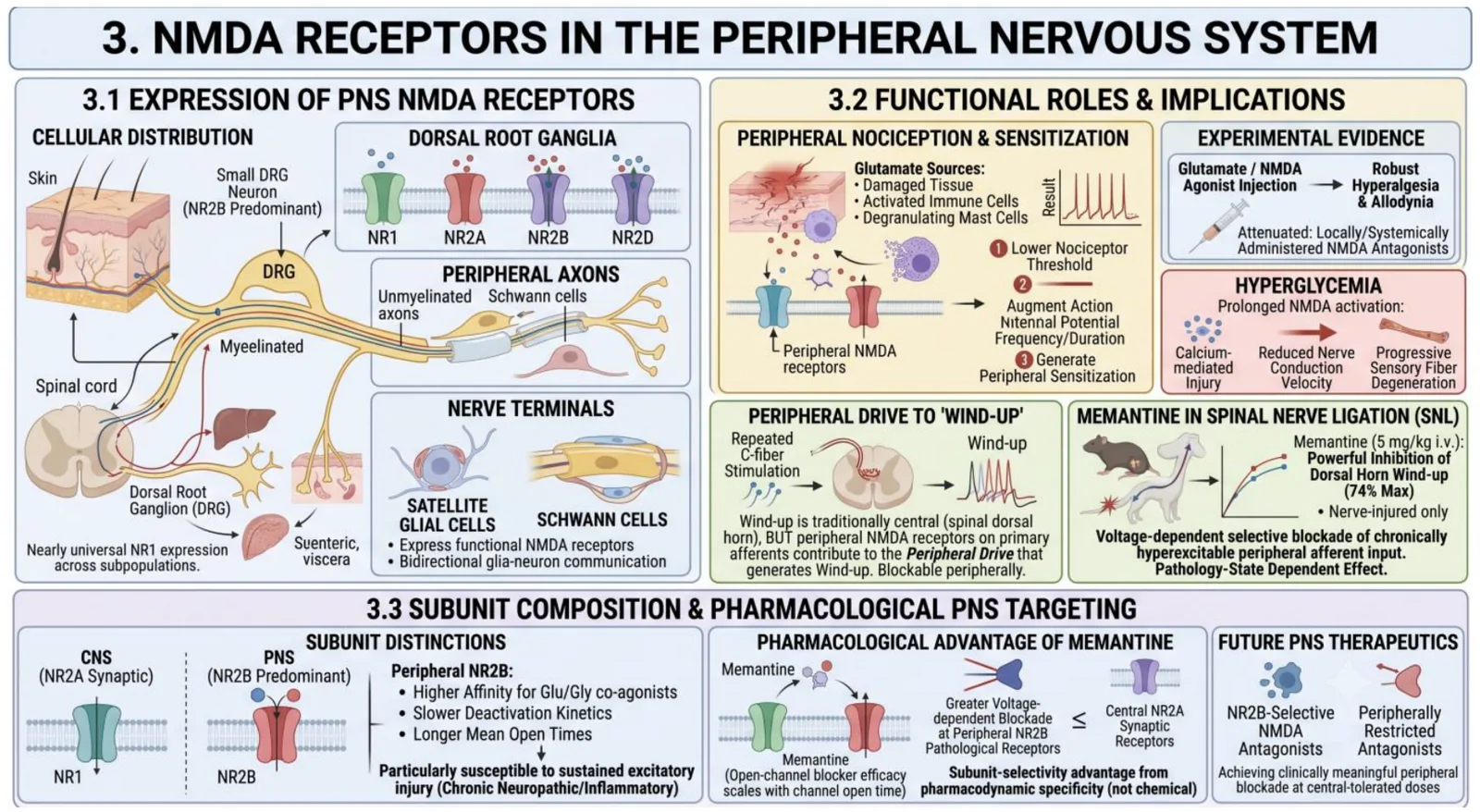
NMDA receptors in the peripheral nervous system. NMDA receptors are broadly expressed across the PNS—skin, dorsal root ganglia (NR2B-predominant small neurons), peripheral axons, nerve terminals, and enteric/visceral sites—with near-universal NR1 expression including in satellite glial and Schwann cells (3.1). Glutamate released from damaged tissue, immune cells, and mast cells activates peripheral NMDA receptors to lower nociceptor threshold, increase firing, and drive peripheral sensitization; agonist injection produces antagonist-reversible hyperalgesia and allodynia, while hyperglycemia-driven activation causes calcium injury, slowed conduction, and fiber degeneration. These receptors contribute a peripheral drive to wind-up, which memantine (5 mg/kg i.v.) inhibits by 74% selectivity in nerve-injured states, reflecting voltage- and pathology-dependent blockade (3.2). The PNS-predominant NR2B subunit—with higher co-agonist affinity, slower deactivation, and longer open times—is especially vulnerable to chronic excitatory injury and confers upon memantine a pharmacodynamic selectivity advantage, supporting future NR2B-selective and peripherally restricted antagonists effective at central-tolerated doses (3.3).

The existence of functional NMDA receptors in the PNS is no longer in doubt. Immunohistochemical, in situ hybridization, and electrophysiological studies have demonstrated the expression of NR1 (GluN1), NR2A (GluN2A), NR2B (GluN2B), and NR2D (GluN2D) subunits in dorsal root ganglion neurons, peripheral unmyelinated and myelinated axons, and peripheral nerve terminals in skin, muscle, and viscera [6,7,18]. NR1 subunit expression is nearly universal across DRG neuron subpopulations, including both large-diameter Aβ mechanoreceptive afferents and small-diameter peptidergic and non-peptidergic C-fiber nociceptors. NR2B subunit expression predominates in small DRG neurons, consistent with their higher sensitivity to sustained NMDA receptor activation and their role in nociceptive sensitization [19] (Figure 2).

Beyond neuronal populations, excitatory amino acid receptors, including NMDA receptor subunits, are expressed on Schwann cells, satellite glial cells of sensory ganglia, sympathetic postganglionic neurons, and enteric nervous system neurons [7,20]. Satellite glial cells, which intimately envelop DRG neuronal cell bodies, express functional NMDA receptors that participate in bidirectional glia–neuron communication and contribute to sensitization of the ganglion during peripheral inflammation and nerve injury [20]. The discovery of perineuronal and Schwann cell NMDA receptor expression broadens the cellular landscape within which peripheral NMDA receptor-targeting drugs like memantine could exert neuroprotective effects (Figure 2).

### 3.2. Functional Roles in Peripheral Nociception and Sensitization

In the peripheral nociceptive system, glutamate, released from damaged or inflamed tissues, activated immune cells, and degranulating mast cells, acts on peripheral NMDA receptors to lower nociceptor activation thresholds, augment the frequency and duration of action potential discharge, and generate peripheral sensitization [6,18]. The intraplanar injection of glutamate or NMDA agonists produces robust hyperalgesia and allodynia in rodents, and these effects are attenuated by locally or systemically administered NMDA antagonists, directly demonstrating the functional relevance of peripheral NMDA receptor activation to pain [7]. In hyperglycemia, prolonged NMDA receptor activation in peripheral neurons of the dorsal horn leads to calcium-mediated injury, reduced nerve conduction velocity, and progressive sensory fiber degeneration [9] (Figure 2).

Wind-up—the progressive amplification of nociceptive responses to repeated C-fiber stimulation—has long been considered a predominantly spinal dorsal horn phenomenon mediated by NMDA receptors on postsynaptic neurons. However, peripheral NMDA receptors on primary afferents contribute to the peripheral drive that generates wind-up, and this peripheral component can be selectively blocked by peripherally administered NMDA antagonists [8]. In the spinal nerve ligation model of peripheral neuropathy, memantine produces powerful inhibition of dorsal horn wind-up specifically in nerve-injured animals (74% maximal inhibition at 5 mg/kg i.v., *p* < 0.02 versus sham), consistent with voltage-dependent selective blockade of chronically hyperexcitable peripheral afferent input [8]. This model directly demonstrates that the peripheral pathological state, rather than the drug dose, determines the magnitude of memantine’s electrophysiological effect (Figure 2).

### 3.3. Subunit Composition and Pharmacological Implications for PNS Targeting

The predominance of NR2B-containing NMDA receptors in peripheral sensory neurons has important pharmacological implications [19]. NR2B-containing receptors display higher affinity for glutamate and glycine co-agonists, slower deactivation kinetics following glutamate removal, and longer mean open times compared to NR2A-dominant synaptic receptors in the CNS. These properties render peripheral NMDA receptors particularly susceptible to sustained excitatory injury during chronic neuropathic or inflammatory conditions, and potentially more sensitive to open-channel blockers such as memantine, whose blocking efficacy scales with channel open time [2,19]. The implication is that memantine may exert a relatively greater voltage-dependent blockade at peripheral NR2B-containing receptors under pathological conditions than at central NR2A-dominant synaptic receptors—a subunit-selective advantage arising from pharmacodynamic rather than chemical specificity (Figure 2).

This mechanistic distinction motivates the development of NR2B-selective or peripherally restricted NMDA antagonists as next-generation therapeutic agents for peripheral neuropathic conditions. However, it also implies that memantine itself, despite its modest NR2B subunit selectivity, may achieve clinically meaningful peripheral NMDA receptor blockade at doses that are tolerated at central NR2A-containing receptors—the pharmacokinetic and pharmacodynamic basis for this hypothesis warrants systematic investigation [19,21] (Figure 2).

## 4. Mechanisms of Memantine’s Protective Actions in PNS

### 4.1. Attenuation of Excitotoxic Calcium Influx in Peripheral Neurons

Pathological NMDA receptor activation is the proximal cause of excitotoxic calcium entry in peripheral neurons exposed to sustained glutamatergic drive, whether arising from hyperglycemia, chemotherapy-induced glutamate dysregulation, ischemia–reperfusion, or nerve-injury-associated neuroinflammation [17,22]. NMDA receptor channels exhibit high calcium permeability (PCa/PNa ~10) and, under conditions of prolonged channel opening, intracellular free calcium rises beyond the homeostatic buffering capacity of calbindin, parvalbumin, and mitochondrial calcium uniporters, triggering downstream activation of calcium-dependent proteases (calpain-1, calpain-2), phospholipases A2 and C, calcineurin, nitric oxide synthase (nNOS), and endonucleases [22,23]. This cascade produces axonal cytoskeletal degradation, myelin membrane peroxidation, mitochondrial outer membrane permeabilization (MOMP), and ultimately apoptosis or necroptosis.

**Figure 3:**
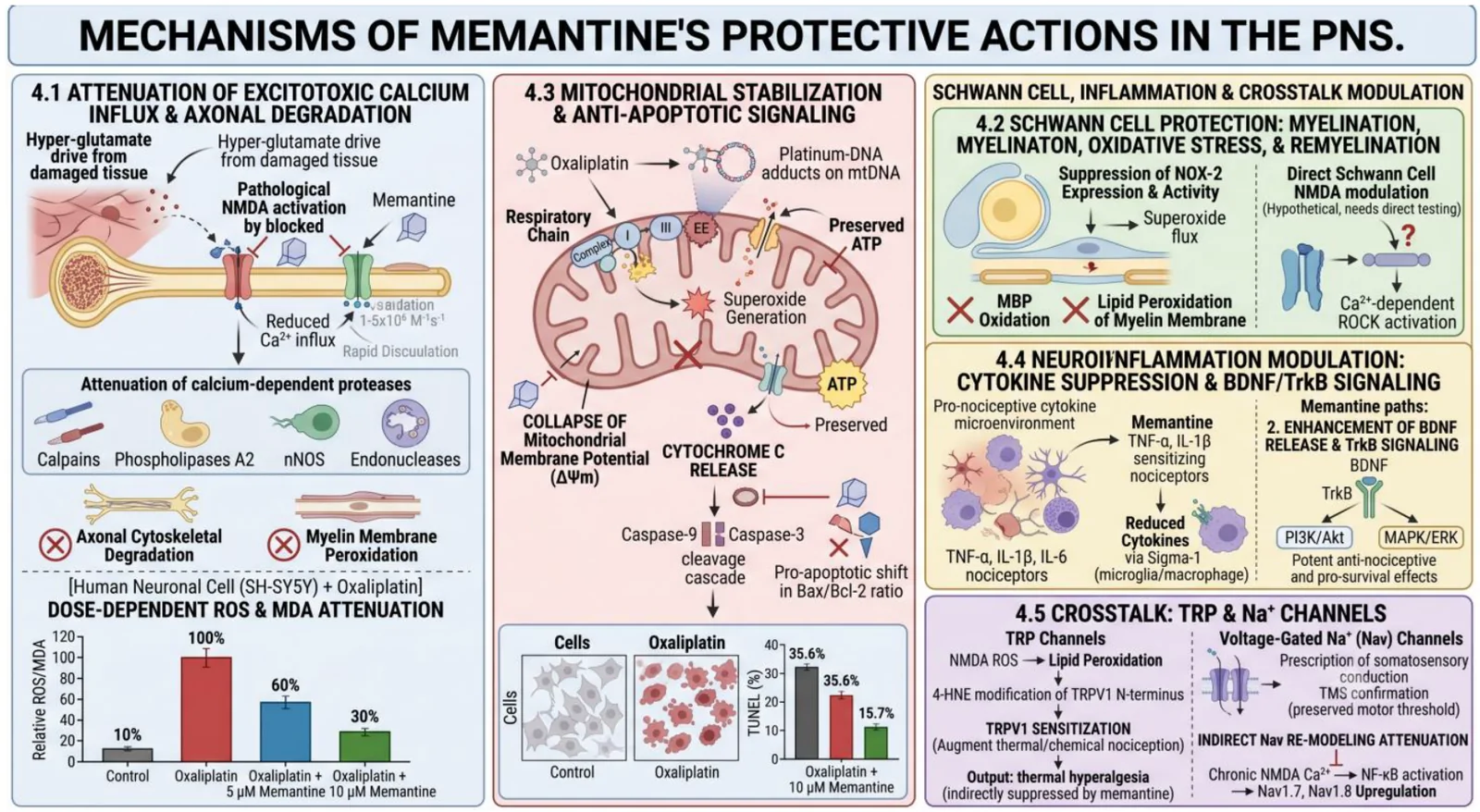
Mechanisms of memantine’s protective actions in the PNS. By blocking pathological NMDA receptor activation from hyperglutamate release, memantine reduces excitotoxic Ca^2+^ influx and calcium-dependent protease activity (calpains, PLA2, nNOS, endonucleases), limiting axonal degradation and myelin peroxidation; in oxaliplatin-treated SH-SY5Y cells, it dose-dependently lowers ROS/MDA (to 60% at 5 μM, 30% at 10 μM) (4.1). It stabilizes mitochondria by preserving membrane potential and ATP, suppressing cytochrome c–driven caspase apoptosis and the Bax/Bcl-2 shift (4.3); protects Schwann cells by reducing NOX-2 activity, MBP oxidation, and myelin lipid peroxidation (4.2); dampens neuroinflammation via sigma-1-mediated cytokine suppression (TNF-α, IL-1β, IL-6) and enhanced BDNF/TrkB signaling (4.4); and modulates ion channel crosstalk by indirectly suppressing TRPV1 sensitization and attenuating NF-κB-driven upregulation of Naᵥ1.7/Naᵥ1.8 while preserving motor conduction (4.5).

Memantine interrupts this cascade at its initiating step by reducing NMDA receptor-mediated Ca^2+^ influx in a voltage-dependent, pathology-selective manner. In oxaliplatin-treated SH-SY5Y human neuronal cells, memantine at 5 and 10 μM significantly attenuated the elevation of intracellular ROS and malondialdehyde (MDA), an index of lipid peroxidation, downstream of calcium-activated phospholipase A2 in a dose-dependent fashion [17]. These in vitro findings are consistent with the established mechanism of memantine-mediated Ca^2+^ attenuation in central neurons and provide direct evidence for its operation in a human peripheral neuronal cell line exposed to a clinically relevant neurotoxic stimulus (Figure 3).

### 4.2. Schwann Cell Protection: Myelination, Oxidative Stress, and Remyelination

Schwann cells are the primary myelinating cells of the PNS and are essential for axonal trophic support, saltatory conduction, and nerve regeneration after injury. They express NMDA receptor subunits (particularly NR1 and NR2B) and are susceptible to glutamate-mediated oxidative injury [7,20]. Oxidative stress generated by NOX-2-derived superoxide anions and downstream hydroxyl radicals causes oxidative modification of myelin basic protein (MBP), peroxidation of phosphatidylcholine and sphingomyelin in the myelin membrane, and disruption of the lipid–protein architecture required for compact myelin maintenance [17,24]. These processes occur in diabetic neuropathy, CIPN, and traumatic nerve injury, contributing to paranodal demyelination and slowing of nerve conduction velocity (Figure 3).

Memantine’s suppression of NOX-2 expression and activity, demonstrated in oxaliplatin-treated neuronal models, reduces the flux of superoxide into the peri-axonal space where it can access myelin-associated lipids [17]. By attenuating oxidative stress at its source, memantine indirectly protects the Schwann cell–myelin unit from peroxidative degradation. Whether memantine also directly modulates Schwann cell NMDA receptor signaling, and whether this modulation promotes remyelination after injury by supporting Schwann cell survival and dedifferentiation, remains an important question for future investigation. Preliminary evidence that NMDA receptor activation in Schwann cells promotes a pro-demyelinating phenotype through Ca^2+^-dependent Rho kinase (ROCK) activation suggests that NMDA receptor blockade could support remyelination, but this hypothesis requires direct experimental testing [24].

### 4.3. Mitochondrial Stabilization and Anti-Apoptotic Signaling

Mitochondrial dysfunction is a cardinal feature of multiple peripheral neuropathy types and represents a mechanistically proximal target for neuroprotective intervention. In the oxaliplatin model of CIPN, platinum–DNA adducts in mitochondrial DNA (which lacks the protective nucleosomal organization of nuclear DNA) impair respiratory chain Complex I and Complex III function, dramatically increasing electron leakage and superoxide generation at the inner mitochondrial membrane [17,25]. This mitochondrial oxidative stress collapses the mitochondrial membrane potential (ΔΨm), reduces ATP synthesis, promotes cytochrome C release into the cytosol, and activates the intrinsic apoptotic cascade through caspase-9 and caspase-3 cleavage.

Memantine treatment in oxaliplatin-exposed SH-SY5Y neurons significantly reversed these apoptotic indices in a dose-responsive manner [17]. Specifically, memantine restored ΔΨm towards control levels, preserved ATP production, reduced cytochrome C cytosolic release, attenuated the pro-apoptotic shift in the Bax/Bcl-2 ratio, and decreased the proportion of TUNEL-positive (apoptotic) cells from 35.6% (oxaliplatin alone) to 15.7% (oxaliplatin + 10 μM memantine) [17]. These findings indicate that memantine’s mitochondrial stabilization is not secondary to its calcium-attenuating effects alone but may involve direct interactions with mitochondrial membranes or oxidative stress pathways, though the precise molecular mechanism of this mitochondrial protection warrants further investigation.

### 4.4. Neuroinflammation Modulation: Cytokine Suppression and BDNF/TrkB Signaling

Neuroinflammation is a central pathogenic mechanism in peripheral neuropathy, involving activated macrophages, mast cells, satellite glial cells, and T lymphocytes that collectively sustain a pro-nociceptive cytokine microenvironment within injured peripheral nerve tissue [26]. Pro-inflammatory cytokines, including tumor necrosis factor-alpha (TNF-α), interleukin-1β (IL-1β), and interleukin-6 (IL-6), sensitize nociceptors through direct modulation of sodium channel, TRP channel, and NMDA receptor expression and trafficking, and through promotion of arachidonic-acid-derived inflammatory mediator release [26,27].

Memantine has been associated with significant reductions in pro-inflammatory cytokine levels across multiple clinical and experimental contexts, including bipolar disorder, depression, and animal models of neuroinflammation [28]. The drug’s anti-inflammatory mechanism is partially independent of NMDA receptor blockade, likely involving sigma-1 receptor modulation of microglial and macrophage activation, and may additionally operate through enhancement of brain-derived neurotrophic factor (BDNF) release and TrkB receptor signaling [28]. BDNF exerts potent anti-nociceptive and pro-survival effects on peripheral sensory neurons through TrkB-activated PI3K/Akt and MAPK/ERK pathways, and its depletion in DRG neurons contributes to neuropathic pain states [29]. The BDNF-enhancing property of memantine may therefore provide a complementary neuroprotective mechanism operating in parallel to its NMDA receptor-mediated actions (Figure 3).

### 4.5. Crosstalk with TRP Channels, Voltage-Gated Sodium Channels, and Ion Channel Remodeling

Peripheral nociceptor sensitization involves complex cross-regulatory interactions between NMDA receptors, transient receptor potential (TRP) channels, and voltage-gated sodium channels (Nav) that collectively determine membrane excitability, the action potential threshold, and stimulus–response gain [18,30]. Oxidative stress downstream of NMDA receptor activation promotes direct lipid peroxidation-mediated TRPV1 sensitization through 4-hydroxynonenal (4-HNE) covalent modification of cysteine residues in the TRPV1 N-terminus, augmenting thermal and chemical nociception [30]. By reducing ROS flux, memantine may indirectly suppress TRPV1 sensitization and the associated thermal hyperalgesia characteristic of CIPN and DPN.

At therapeutic concentrations, memantine does not directly block Nav channels. This is supported by TMS studies demonstrating that the motor threshold remained unchanged after 8 days of 30 mg/day memantine, confirming the preservation of Nav-mediated membrane excitability [14]. This selectivity is advantageous for peripheral analgesic applications, as Nav blockade would impair normal somatosensory conduction. Conversely, the Nav1.7 and Nav1.8 upregulation that occurs in DRG neurons following peripheral nerve injury and NMDA receptor activation may be indirectly attenuated by memantine through reduction of the calcium-dependent transcription factor NF-κB activation that drives Nav subtype remodeling in neuropathic states [18,30] (Figure 3).

## 5. Memantine in Peripheral Neuropathies of the Somatic Nervous System (Sensory and Motor Divisions)

**Figure 4:**
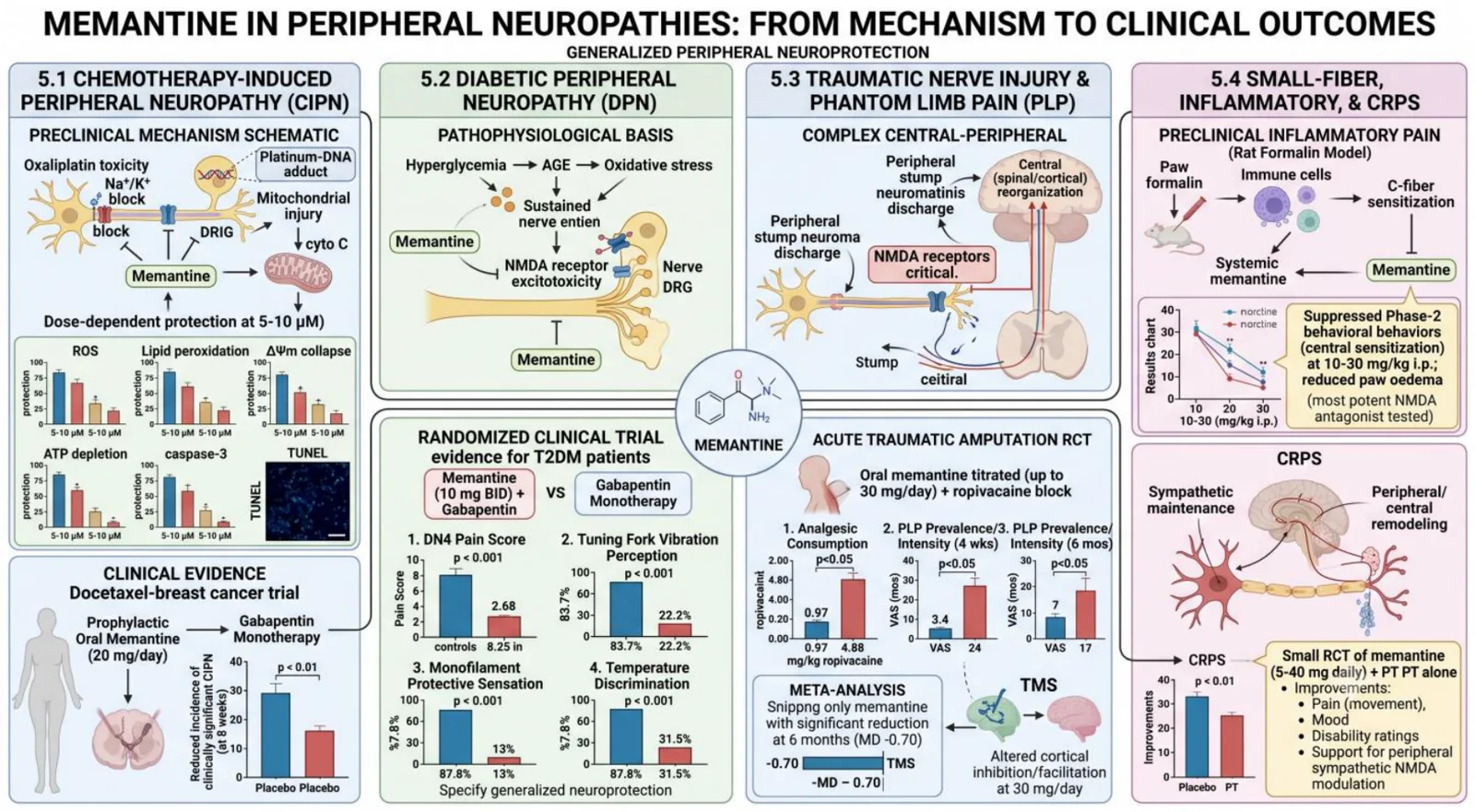
Memantine in peripheral neuropathies: from mechanism to clinical outcomes. In chemotherapy-induced peripheral neuropathy (CIPN), memantine counters oxaliplatin toxicity and mitochondrial injury with dose-dependent protection (5–10 μM) against ROS, lipid peroxidation, ΔΨm collapse, ATP depletion, and caspase-3 apoptosis; a docetaxel breast-cancer trial showed reduced clinically significant CIPN with prophylactic oral memantine (20 mg/day) (5.1). In diabetic peripheral neuropathy (DPN), memantine interrupts the hyperglycemia–AGE–oxidative stress–NMDA excitotoxicity cascade, and a T2DM RCT (10 mg BID + gabapentin) improved DN4 pain, vibration perception, protective sensation, and temperature discrimination (5.2). In traumatic nerve injury and phantom limb pain (PLP), where peripheral neuroma discharge and central reorganization both engage NMDA receptors, an amputation RCT (memantine up to 30 mg/day + ropivacaine) reduced analgesic use and PLP prevalence/intensity, with meta-analysis showing significant pain reduction at 6 months (MD −0.70) (5.3). In small-fiber, inflammatory, and CRPS conditions, systemic memantine suppressed phase-2 formalin behaviors and paw edema (10–30 mg/kg), and a small CRPS RCT (5–40 mg/day + PT) improved pain, mood, and disability, supporting peripheral sympathetic NMDA modulation (5.4).

Following the functional division of the peripheral nervous system, Section 5 addresses conditions of the somatic nervous system—both the sensory afferent limb (chemotherapy-induced peripheral neuropathy, diabetic peripheral neuropathy, small-fiber and inflammatory neuropathies) and the motor efferent limb encountered through central-peripheral loops of amputation-related pain (phantom limb pain) and complex regional pain syndrome. Autonomic nervous system involvement—including the sympathetic, parasympathetic, and enteric divisions, as well as the neuromuscular junction as the somatic motor terminal synapse—is treated separately in Section 6, and cardiovascular, gastrointestinal, and renal end-organ effects downstream of autonomic and vascular NMDA receptor signaling are treated in Section 7 (Figure 4).

### 5.1. Chemotherapy-Induced Peripheral Neuropathy (CIPN)

Chemotherapy-induced peripheral neuropathy is one of the most clinically significant dose-limiting toxicities of cancer treatment, affecting 30–70% of patients receiving platinum compounds, taxanes, vinca alkaloids, or bortezomib, and persisting as a long-term sequela in a substantial proportion [25,31]. CIPN most commonly manifests as a length-dependent, predominantly sensory polyneuropathy characterized by paresthesia, dysesthesia, allodynia, and proprioceptive impairment, with a neurobiological basis in DRG neuron injury, intraepidermal nerve fiber (IENF) loss, and dorsal horn sensitization. No agent has yet received regulatory approval for CIPN prevention or treatment, representing a major unmet clinical need.

The mechanistic rationale for memantine in CIPN is compelling. Oxaliplatin induces acute peripheral neurotoxicity through direct inhibition of voltage-gated Na^+^ and K^+^ channels in peripheral axons and chronic neurotoxicity through platinum–DNA adduct-mediated mitochondrial injury in DRG neurons [25]. Both components generate excess intracellular calcium and ROS, activating the excitotoxic and apoptotic cascades that memantine attenuates. Direct experimental evidence from the SH-SY5Y neuronal cell model demonstrates dose-dependent memantine protection against oxaliplatin-induced ROS elevation, lipid peroxidation, NOX-2 upregulation, ΔΨm collapse, ATP depletion, cytochrome C release, Bax/Bcl-2 shift, caspase-3 activation, and TUNEL-positive apoptosis at 5–10 μM [17]. Two important caveats limit the direct translation of this in vitro dataset. First, SH-SY5Y neuroblastoma cells are a catecholaminergic tumor line that only partially recapitulates the molecular and functional phenotype of primary dorsal root ganglion neurons or Schwann cells, and their NMDA receptor complement and calcium-handling machinery may not fully match adult peripheral sensory neurons. Second, the 5–10 μM concentrations used in vitro exceed steady-state free plasma concentrations achievable at conventional oral doses by roughly one order of magnitude, and no direct peripheral-nerve K_p_ data are yet available. The SH-SY5Y results should therefore be interpreted as a mechanistic proof-of-concept rather than as direct evidence that clinically achievable exposures reproduce the same protection in vivo. These findings provide a mechanistic foundation for clinical evaluation.

At the clinical level, a randomized clinical trial in 40 women with non-metastatic breast cancer receiving AC-T (with docetaxel) chemotherapy demonstrated that prophylactic oral memantine (20 mg/day, commenced with the first cycle of docetaxel and continued for eight weeks) significantly reduced the DN4 score and shortened neuropathy duration compared with the untreated control group, with 40% of memantine-treated patients remaining neuropathy-free versus 10% of controls [32]. Translation of the preclinical mechanistic findings to adequately powered multi-site RCTs evaluating CIPN prevention across oxaliplatin-based colorectal cancer regimens and taxane-based breast cancer regimens represents a priority research direction with immediate clinical relevance.

### 5.2. Diabetic Peripheral Neuropathy (DPN)

Diabetic peripheral neuropathy affects 30–50% of individuals with diabetes mellitus and constitutes the most prevalent peripheral neuropathy globally, contributing enormously to patient morbidity through painful neuropathic symptoms, foot ulcerations, and lower-extremity amputation [33]. The pathophysiological basis of DPN is multifactorial, encompassing hyperglycemia-induced polyol pathway activation, advanced glycation end-product (AGE) accumulation, protein kinase C activation, oxidative and nitrosative stress, impaired neurotrophic support, and, importantly, sustained NMDA receptor-mediated excitotoxicity in peripheral and dorsal horn neurons [9,33]. The persistent activation of NMDA receptors in peripheral neurons by diabetes-associated glutamate dyshomeostasis generates calcium-mediated axonal injury that is mechanistically addressable by memantine.

A randomized clinical trial enrolling 143 patients with confirmed type 2 diabetes mellitus and DPN compared memantine (5 mg twice daily, titrated to 10 mg twice daily) combined with gabapentin (300 mg daily) versus gabapentin monotherapy over an 8-week treatment period [9]. The memantine combination group demonstrated significantly superior outcomes across all assessed neuropathy domains. The Douleur Neuropathique 4 (DN4) score, a validated neuropathic pain screening instrument, fell from a mean of 9.1 at baseline to 2.68 at the end of the 8 weeks in the memantine group versus 8.25 in controls (*p* < 0.001). The tuning fork vibration perception test improved to 83.7% in the memantine group versus 22.2% in controls (*p* < 0.001); monofilament protective sensation testing improved to 87.8% versus 13% (*p* < 0.001); and temperature discrimination testing improved to 87.8% versus 31.5% (*p* < 0.001). The magnitude and breadth of these improvements spanning neuropathic pain scores, vibration sensation, pressure sensation, and thermal discrimination indicate that memantine’s effect operates across multiple peripheral sensory modalities, consistent with a mechanism of generalized peripheral neuroprotection rather than symptom-specific analgesia [9]. It should be emphasized that the 8-week DPN trial by Jafarzadeh et al. assessed changes in functional sensory thresholds (DN4, monofilament, tuning fork, and tip-therm testing) rather than structural indices of peripheral nerve integrity such as intraepidermal nerve fiber density, nerve conduction studies, or corneal confocal microscopy. Improvements in these questionnaire- and bedside-based endpoints therefore reflect symptomatic and functional benefits and should not be interpreted as direct evidence of structural nerve regeneration or reversal of axonopathy.

### 5.3. Traumatic Nerve Injury and Phantom Limb Pain

Phantom limb pain (PLP) and phantom limb sensations occur in 50–78% of patients following limb amputation and represent a complex central–peripheral sensitization phenomenon in which altered cortical reorganization, spinal dorsal horn sensitization, and peripheral stump neuroma discharge interact to generate persistent pain in the absent limb [16,34]. NMDA receptors are critically implicated in the deafferentation-induced membrane potential changes and long-term potentiation in spinal and supraspinal circuits that underlie PLP, and pre-emptive NMDA receptor blockade before the establishment of central sensitization has been proposed as a pharmacological strategy for its prevention [16].

A landmark double-blind RCT by Maier et al. [16] enrolled 36 patients (18 per group) with chronic phantom limb pain following limb amputation and randomized them to receive oral memantine (titrated from 10 mg/day in week 1 to 30 mg/day in weeks 3–4) or a placebo for four weeks, in conjunction with continuous brachial plexus blockade with ropivacaine 0.375% [16]. Memantine treatment significantly reduced the volume of analgesic consumed (0.97 mg/kg ropivacaine boluses versus 4.88 mg/kg in placebo, *p* < 0.05) and produced significantly lower PLP prevalence and intensity at four weeks (VAS 3.4 versus 24, *p* < 0.05) and six months (VAS 7 versus 17, *p* < 0.05), though these differences were not sustained at 12-month follow-up. It should be emphasized that, given the small sample size, the trial was underpowered for several secondary endpoints, and any non-significant contrasts should be interpreted as inconclusive rather than as evidence of no effect (Type II error risk). Furthermore, because systemic memantine crosses the blood–brain barrier, the observed analgesic benefit likely reflects convergent action at peripheral stump neuromas and at central (spinal and cortical) sites and cannot be attributed exclusively to peripheral NMDA receptor blockade. A systematic review and meta-analysis of perioperative pharmacological interventions for PLP identified memantine as the only agent demonstrating any statistically significant pooled reduction in PLP intensity at six months (MD −0.70, 95% CI −0.18 to −1.23), though with low certainty of evidence due to limited sample sizes [34].

TMS studies conducted in healthy volunteers after 8 days of oral memantine at 30 mg/day demonstrated significant enhancement of intracortical inhibition and reduction in intracortical facilitation changes that correlated inversely with serum memantine concentrations, suggesting that memantine modulates the cortical plasticity mechanisms that participate in post-amputation sensorimotor reorganization [14]. These central effects, operating in parallel with peripheral NMDA receptor blockade at the stump neuroma and residual afferent fibers, may collectively underlie memantine’s prophylactic efficacy against PLP [14,16].

### 5.4. Small-Fiber Neuropathy, Inflammatory Neuropathy, and Complex Regional Pain Syndrome

In inflammatory and small-fiber neuropathies, peripheral glutamate release from activated immune cells, damaged epithelium, and degranulating mast cells produces C-fiber sensitization through peripheral NMDA receptor activation that amplifies afferent discharge and lowers pain thresholds [6,18]. In a rat formalin model of acute inflammatory pain, systemic memantine suppressed late-phase (phase-2) biting and licking behaviors attributed to central sensitization driven by peripheral C-fiber input at doses of 10–30 mg/kg i.p., with memantine being the most potent of three NMDA antagonists tested (dextromethorphan, memantine, ketamine) for phase-2 suppression and reduction in formalin-induced paw edema [10].

Complex regional pain syndrome (CRPS) represents a peripheral and central sensitization disorder in which sympathetically maintained pain, vasomotor dysfunction, and inflammatory changes in the affected extremity are driven by convergent peripheral NMDA receptor upregulation and central synaptic remodeling [35]. A 20-patient RCT in CRPS evaluated memantine (5–40 mg/day, escalated over 49 days) combined with physiotherapy versus physiotherapy alone, demonstrating that the memantine group but not controls showed significant improvements in pain with movement, mood, and disability ratings [35]. These findings should, however, be considered hypothesis-generating rather than confirmatory: with only 10 patients per arm, the trial is underpowered and vulnerable to Type II error, and the fMRI data reported by Gustin et al. showed that the combination of morphine plus memantine reduced pain-evoked activation in contralateral primary and secondary somatosensory cortices, so the observed analgesic benefit clearly engages central (cortical) pathways in addition to any peripheral sympathetic NMDA receptor modulation. Attributing the response predominantly to peripheral NMDA blockade would therefore over-interpret the available evidence. These findings nevertheless support the clinical relevance of peripheral sympathetic NMDA receptor modulation in CRPS as one component of a convergent peripheral–central mechanism, though the small sample size and the lack of a pharmacological-only control arm limit conclusions about the independent contribution of memantine versus physiotherapy.

## 6. Memantine at the Somatic Neuromuscular Junction and in the Autonomic Nervous System (Sympathetic, Parasympathetic, and Enteric Divisions)

Section 6.1 addresses the somatic motor terminal synapse (neuromuscular junction) and its relevance to organophosphate-induced neuromuscular failure. Section 6.2 addresses the sympathetic and parasympathetic divisions of the autonomic nervous system, focused on cervical, celiac, and other autonomic ganglia. Enteric nervous system pharmacology, historically considered the third division of the autonomic nervous system, is addressed within Section 7.2 in the context of gastrointestinal end-organ effects.

### 6.1. Neuromuscular Junction Pharmacology and Protection Against Organophosphate Toxicity

The neuromuscular junction (NMJ) is the primary peripheral synapse through which the motor nervous system controls skeletal muscle contraction, and its function depends critically on the fidelity of acetylcholine (ACh) release, nicotinic AChR activation, and acetylcholinesterase (AChE)-mediated ACh hydrolysis. Memantine interacts with the NMJ through two pharmacologically distinct mechanisms: open-channel blockade of the nicotinic receptor–sodium ionophore complex and, at higher concentrations, AChE inhibition [13]. These properties are relevant to the application of memantine as a prophylactic agent against organophosphate (OP) nerve agent toxicity, where irreversible AChE inhibition causes ACh accumulation, sustained NMJ depolarization, depolarization block of neuromuscular transmission, and respiratory failure.

In soman-poisoned rats, memantine pretreatment at 18 mg/kg i.m. significantly protected neuromuscular transmission as assessed by the phrenic nerve–diaphragm preparation in situ, attenuating the reduction in diaphragmatic contractile amplitude following soman challenge with a faster onset of protection than physostigmine or pyridostigmine [13]. Importantly, memantine at the prophylactic dose did not itself reduce baseline contractility, distinguishing an advantage over carbamate AChE inhibitor prophylactics, which pre-emptively suppress NMJ function and thereby reduce diaphragmatic reserve. Memantine’s nicotinic receptor–sodium ionophore blockade mechanism directly counteracts the ACh-induced depolarization block that is the immediate cause of neuromuscular respiratory failure following OP exposure [13].

However, the narrow therapeutic index of memantine in this context is a significant limitation: the effective prophylactic dose represents 12.22% of the LD50 compared to 2.98% for pyridostigmine, implying substantially less margin between protection and toxicity [13]. This finding indicates that NMJ-protective applications of memantine require precise dose titration and monitoring, and that peripherally restricted formulations with reduced CNS penetration may be necessary to improve the therapeutic window in organophosphate prophylaxis contexts.

### 6.2. Autonomic Ganglia Modulation and Dysautonomia

The autonomic nervous system communicates through ganglionic synapses that employ both nicotinic ACh receptors and NMDA receptors as co-transmitters, with NMDA receptor activation in superior cervical and celiac ganglia capable of potentiating autonomic ganglionic transmission and modulating sympathetic outflow [36]. In diabetic autonomic neuropathy, the most common form of autonomic dysfunction globally—pathological glutamate accumulation in autonomic ganglia—has been implicated in the progressive degeneration of sympathetic and parasympathetic postganglionic neurons that underlies cardiovascular autonomic neuropathy, erectile dysfunction, gastroparesis, and orthostatic hypotension [33,36].

Direct evidence for memantine’s effects on autonomic ganglia NMDA receptor function in intact neural circuits is limited, but the mechanistic basis for its activity is established by the demonstration of functional NMDA receptors in autonomic ganglia and by the extrapolation from DRG and dorsal horn neuroprotection models. The exclusion of autonomic neuropathy as a comorbidity in the DPN RCT by Jafarzadeh et al. prevents assessment of autonomic effects in that dataset: dedicated clinical trials enrolling patients with cardiovascular autonomic neuropathy or diabetic gastroparesis, incorporating established autonomic function endpoints (heart rate variability, tilt-table testing, gastric emptying scintigraphy), are warranted [9,36].

## 7. Memantine in Cardiovascular, Gastrointestinal, and Renal Systems

### 7.1. Cardiovascular System

NMDA receptors are expressed in vascular endothelial cells and vascular smooth muscle cells, where they participate in the regulation of vascular tone, endothelial nitric oxide synthase (eNOS) activity, and endothelial barrier function [37]. Glutamate-induced excitotoxicity in cerebrovascular and peripheral vascular endothelium involves NMDA receptor-mediated Ca^2+^ influx, subsequent nNOS/eNOS overactivation, peroxynitrite generation, mitochondrial uncoupling, and cell death—a process mechanistically analogous to neuronal excitotoxicity [22,37]. Peripheral vascular NMDA receptor activation has been implicated in endothelial dysfunction in the context of hyperglycemia and ischemia–reperfusion, potentially contributing to the microvascular pathology of diabetic complications.

**Figure 5:**
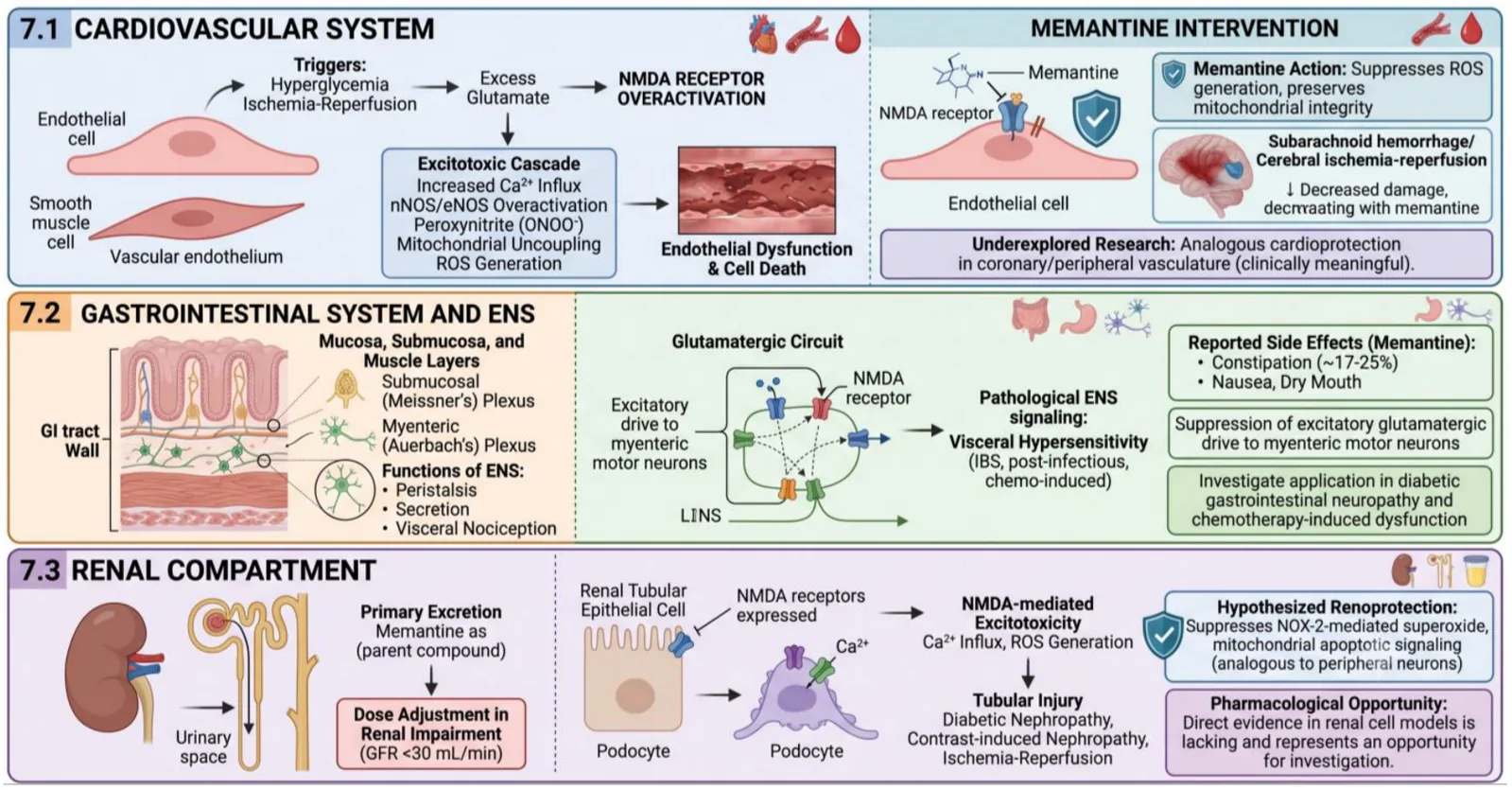
Memantine in cardiovascular, gastrointestinal, and renal systems. In the cardiovascular system, triggers such as hyperglycemia and ischemia–reperfusion drive excess glutamate and NMDA receptor overactivation, producing an excitotoxic cascade (Ca^2+^ influx, nNOS/eNOS overactivation, peroxynitrite, mitochondrial uncoupling, ROS) that causes endothelial dysfunction and cell death; memantine suppresses ROS and preserves mitochondrial integrity, decreasing damage in subarachnoid hemorrhage and cerebral ischemia–reperfusion, with analogous coronary/peripheral cardioprotection underexplored (7.1). In the gastrointestinal tract and enteric nervous system, NMDA receptors within the submucosal and myenteric plexuses regulate peristalsis, secretion, and visceral nociception; their pathological signaling underlies visceral hypersensitivity (IBS, post-infectious, chemo-induced), and memantine suppresses excitatory glutamatergic drive to myenteric motor neurons—though with GI side effects (constipation ~17–25%, nausea, dry mouth)—suggesting potential in diabetic GI neuropathy and chemotherapy-induced dysfunction (7.2). In the renal compartment, memantine is excreted largely unchanged (requiring dose adjustment at GFR <30 mL/min), and NMDA receptors on tubular epithelial cells and podocytes mediate Ca^2+^/ROS excitotoxic tubular injury in diabetic, contrast-induced, and ischemia–reperfusion nephropathy; hypothesized renoprotection via NOX-2 and mitochondrial apoptotic suppression remains to be tested directly (7.3).

In experimental models of subarachnoid hemorrhage and cerebral ischemia–reperfusion, memantine reduces cerebrovascular endothelial injury through suppression of NMDA receptor-mediated ROS generation and preservation of mitochondrial membrane integrity [17,38]. Whether analogous cardioprotective effects operate in the coronary and peripheral vasculature has not been systematically evaluated. Given that cardiovascular autonomic neuropathy and macrovascular disease are major sources of mortality in diabetic patients, and that NMDA receptor antagonism attenuates hyperglycemia-driven endothelial dysfunction in cell culture models, the cardiovascular dimension of memantines peripheral pharmacology represents a clinically meaningful but underexplored research area (Figure 5).

### 7.2. Gastrointestinal System and the Enteric Nervous System

The enteric nervous system (ENS) constitutes the largest division of the peripheral autonomic nervous system, comprising an estimated 200–600 million neurons distributed in the myenteric (Auerbach’s) and submucosal (Meissner’s) plexuses of the gastrointestinal wall [39]. ENS neurons regulate intestinal peristalsis, secretion, mucosal immunity, and visceral nociception through complex glutamatergic and cholinergic circuits in which NMDA receptors participate as critical modulatory components [39]. Pathological ENS glutamatergic signaling has been implicated in the visceral hypersensitivity of irritable bowel syndrome (IBS), post-infectious neuropathy, chemotherapy-induced gastrointestinal dysfunction, and inflammatory bowel disease.

Gastrointestinal adverse effects reported with memantine, including dry mouth, constipation, and nausea, occurring in approximately 17–25% of treated patients in clinical trials, may partly reflect modulation of ENS NMDA receptor-mediated motility pathways, with constipation potentially indicating suppression of excitatory glutamatergic drive to myenteric motor neurons [5,39]. The converse implication is that memantine might attenuate visceral hypersensitivity in conditions characterized by pathological ENS glutamatergic sensitization—a hypothesis that is mechanistically plausible but clinically untested. Given the substantial burden of gastrointestinal neuropathy in diabetic patients and cancer patients receiving neurotoxic chemotherapy, this application warrants investigation (Figure 5).

### 7.3. Renal Compartment: Pharmacokinetic and Cytoprotective Considerations

Memantine is primarily excreted by the kidney as a parent compound, making renal function the principal determinant of drug clearance and requiring dose reduction in patients with impaired renal function (GFR < 30 mL/min) [5]. Renal tubular epithelial cells and podocytes express NMDA receptors and are susceptible to excitotoxic injury in the context of diabetic nephropathy, contrast-induced nephropathy, and ischemia–reperfusion injury conditions in which NMDA receptor-mediated Ca^2+^ influx and ROS generation contribute to tubular injury [22]. Memantine’s established suppression of NOX-2-mediated superoxide generation and mitochondrial apoptotic signaling in peripheral neurons may confer analogous renoprotection in tubular cells under these injury conditions, although direct evidence in renal cell models is lacking and represents a pharmacological opportunity for investigation.

## 8. Memantine in Optic Nerve and Retinal Pathology

### 8.1. Retinal Ganglion Cell Excitotoxicity and Glaucoma

The retinal ganglion cell (RGC) and its axon, which constitutes the optic nerve, represent a peripheral cranial-nerve system in which glutamate excitotoxicity is a well-established mechanism of neurodegeneration. Elevated vitreal glutamate concentrations have been documented in humans with glaucoma, in monkey models of experimental glaucoma, in rabbit models of optic nerve ischemia, and in patients with proliferative diabetic retinopathy [15,40]. Sustained activation of NMDA receptors on RGC dendrites under conditions of elevated intraocular pressure (IOP) or ischemia drives excitotoxic Ca^2+^ accumulation, mitochondrial dysfunction, and apoptotic RGC death through mechanisms that are structurally and functionally identical to those operating in peripheral sensory neurons [40].

In a rabbit model of optic nerve ischemia induced by continuous endothelin-1 delivery to perineural optic nerve tissue, concurrent daily intramuscular memantine (1 mg/kg) completely prevented morphological changes in optic nerve head parameters (cup area, cup depth, and rim volume) over an eight-week observation period (multivariate ANOVA, *p* = 0.78 in memantine-treated versus *p* < 0.0001 in untreated animals) [15]. In DBA/2J mice—a genetic model of pigmentary angle-closure glaucoma—sustained intraperitoneal memantine (5 mg/kg twice daily, seven months) produced a 20% preservation of b-wave amplitude in scotopic electroretinography (*p* < 0.001 versus untreated controls at 10 months), reduced the proportion of eyes with advanced optic nerve neuropathy from 80% to 59%, and decreased photoreceptor damage from 50% to 19% of eyes [40]. These findings from two independent animal models employing distinct glaucomatous pathological mechanisms provide strong convergent evidence for memantine-mediated RGC neuroprotection.

### 8.2. Clinical Evidence in Glaucoma and Translational Challenges

Two large Phase III RCTs (NCT00141882 and NCT00168350) conducted by Allergan assessed oral memantine (low-dose and high-dose arms) versus a placebo in approximately 1100 patients, each with chronic open-angle glaucoma, over a follow-up period of three years, using visual field mean deviation as the primary endpoint [41]. Preliminary results from the first trial suggested a possible benefit in a subset of patients with more advanced baseline visual field loss, but this finding was not replicated in the second trial. The complete datasets from both trials have not been published in peer-reviewed form, limiting independent evaluation of the clinical evidence and constituting a significant gap in the glaucoma neuroprotection literature [41].

Cochrane’s systematic review of neuroprotective agents for glaucoma concluded that there was no convincing evidence for any currently available agent, acknowledging that this conclusion reflected predominantly the absence of adequately designed trials rather than established inefficacy [41]. The translational gap between robust animal model results and inconclusive Phase III trial data in glaucoma likely reflects several factors: the use of visual field mean deviation as an endpoint requires substantial structural RGC loss before functional change becomes detectable; the trial follow-up may have been insufficient to demonstrate a neuroprotective effect on top of IOP-lowering therapy; and patient selection may have not been enriched for those at highest risk of excitotoxic progression. Biomarker-stratified trials employing retinal ganglion cell layer optical coherence tomography (OCT-RGC) and plasma neurofilament light chain (NfL) as surrogate structural endpoints may provide a more sensitive and earlier signal of neuroprotective efficacy.

## 9. Synthesis of Clinical Evidence in Neuropathic Pain Syndromes

### 9.1. Post-Herpetic Neuralgia and Established Neuropathic Pain States

The evidence for memantine in established, chronic neuropathic pain is heterogeneous and predominantly negative. Two RCTs by Eisenberg et al. [42] (*n* = 24, memantine 10–20 mg/day, 5 weeks) and Martin et al. [21] failed to demonstrate significant benefit in established post-herpetic neuralgia (PHN). The Sang et al. [43] placebo-controlled crossover trial in patients with painful diabetic neuropathy and PHN found only modest, non-significant pain reductions with memantine (17.4% mean reduction from baseline in DPN patients) compared to dextromethorphan (33.4%, also non-significant at the dose studied) [21,42]. These findings are consistent with the hypothesis, now supported by mechanistic and clinical convergence across multiple conditions, that NMDA antagonist therapy is substantially more effective when administered prophylactically before the consolidation of central sensitization than therapeutically after established chronic pain states have been present for months to years.

This prophylactic superiority hypothesis receives strong support from the Morel et al. [44] pre-mastectomy trial, in which memantine (5–20 mg/day commencing four weeks before surgery) reduced the incidence of post-mastectomy neuropathic pain at three months from 30% in the placebo group to 5% in the memantine group (*p* = 0.04) and significantly reduced analgesic requirements in the perioperative period [42]. The mechanistic basis for this prophylactic superiority is the prevention of the initial NMDA receptor-dependent synaptic-strengthening events in spinal and supraspinal circuits that translate acute peripheral injury signals into a chronic central sensitization window of vulnerability that exists prior to surgery and in the early post-injury period but is largely closed once established sensitization has remodeled synaptic architecture [1,42].

### 9.2. Ketamine-Relay and Combination Strategies

A multicenter RCT by Martin et al. [21] enrolled 60 patients with refractory neuropathic pain who had previously responded to intravenous (IV) ketamine infusion, randomizing them to 12-week oral maintenance treatments with dextromethorphan (90 mg/day), memantine (20 mg/day), or a placebo to evaluate sustained analgesic benefit. At one month, pain intensity was preserved (did not increase) with dextromethorphan (*p* = 0.53 versus pre-trial NRS) but increased significantly with memantine and the placebo (*p* = 0.04). However, in three months, the three groups did not differ significantly in pain scores, which remained below pre-trial levels in all groups. Importantly, memantine produced specific improvements relative to the placebo in spatial planning cognitive function (Stockings of Cambridge test, *p* = 0.04), quality-of-life vitality, and role-emotional sub-scores (SF-36, *p* < 0.05 at three months). These findings suggest that even where direct analgesic efficacy is modest, memantine may meaningfully improve the cognitive and functional dimensions of the chronic neuropathic pain experience—an outcome domain that is insufficiently captured by pain NRS scores alone [21].

### 9.3. Quantitative Synthesis of Clinical Signals

Across the clinical trial landscape, memantine demonstrates the most consistent and statistically significant benefit in two clinical contexts: diabetic peripheral neuropathy with active treatment (Jafarzadeh et al. RCT, *n* = 143, with favorable multimodal improvements in functional sensory thresholds, which do not, however, constitute direct evidence of structural nerve regeneration) and prophylactic phantom limb pain prevention (Maier et al., *n* = 36; 18 per arm, with significant 4-week and 6-month benefits; meta-analytic pooled MD −0.70 on 10-point NRS) [9,16,35]. The small samples of Maier (*n* = 36) and Gustin (*n* = 20) confer a substantial risk of Type II error, and their positive signals should be considered as hypothesis-generating rather than definitive. Positive signals are also present in complex regional pain syndrome and pre-emptive post-mastectomy pain prevention [34,40]. The consistently negative results in established post-herpetic neuralgia and the mixed signals in the maintenance of analgesia after ketamine relay suggest that the timing of intervention relative to the injury-sensitization chronology is a critical determinant of clinical response—a hypothesis with major implications for trial design and patient selection in future studies (Table 1).

## 10. Limitations of the Current Evidence Base

### 10.1. Absence of PNS-Specific RCT Designs

The most fundamental limitation of the current evidence base is the near-complete absence of clinical trials specifically designed to assess peripheral rather than central neuroprotective effects of memantine. Existing positive trials were designed with symptom-based endpoints (pain scores, functional disability), which do not differentiate between peripheral and central mechanisms of drug action and do not capture structural or functional indices of peripheral nerve health such as IENF density, nerve conduction velocity, corneal confocal microscopy nerve density, or skin biopsy mRNA expression of nociceptor-specific markers. Without such PNS-specific endpoints, the peripheral neuroprotective mechanism that is mechanistically proposed and preclinically demonstrated remains clinically unverified in a direct and rigorous sense.

### 10.2. Heterogeneity of Animal Models and Translational Uncertainty

The animal data supporting peripheral neuroprotection span diverse models: in vitro SH-SY5Y cell culture, spinal nerve ligation in rats, optic nerve ischemia in rabbits, genetic glaucoma in DBA/2J mice, and organophosphate toxicity in rats that employ different species, different neuropathy mechanisms, and different routes and doses of memantine administration. The relevance of each model to specific human peripheral neuropathies is variable, and the translation of neuroprotective efficacy from acute animal models to the chronic, multifactorial human disease contexts in which memantine would be clinically deployed remains uncertain. Importantly, none of the currently available animal models of DPN or CIPN fully recapitulate the temporal dynamics, severity, or treatment context of the clinical conditions they represent.

### 10.3. Pharmacokinetic (PK) and Pharmacodynamic (PD) Knowledge Gaps

The pharmacokinetic characterization of memantine in peripheral nerve compartments is inadequate. Blood–nerve barrier penetration kinetics, peripheral nerve tissue-to-plasma concentration ratios, and the relationship between systemic plasma concentrations and pharmacologically relevant concentrations at DRG neurons and peripheral nerve terminals have not been systematically measured. This absence of peripheral PK/PD data precludes confident dosing recommendations for peripheral neuroprotective applications and limits the ability to interpret negative clinical trials regarding whether they reflect true therapeutic inefficacy or subtherapeutic drug concentrations at the target tissue. Development of validated peripheral microdialysis or nerve-specific pharmacokinetic methodologies would substantially advance the translational framework for memantine in PNS conditions.

### 10.4. Absence of a Molecular-Chaperone (HSP70) Perspective

A further limitation of the present review is that the discussion of memantine’s neuroinflammatory and cytoprotective actions (Section 4.4) does not integrate its interaction with molecular chaperones, and in particular with the 70 kDa heat shock protein family (HSP70/HSPA1A). Independent lines of evidence indicate that memantine and related non-competitive NMDA receptor antagonists induce HSP70 in specific cortical and hippocampal regions of the rodent brain—memantine specifically has been shown to upregulate HSP70 immunoreactivity in the posterior cingulate cortex, retrosplenial cortex, and dentate gyrus following single systemic doses and to regulate HSP70 mRNA and protein expression in a neonatal rat model of cerebral hypoxic–ischemia [45]. Because HSP70 has well-characterized anti-apoptotic, anti-inflammatory, and proteostatic actions that overlap substantially with the calcium-attenuating, mitochondrial stabilization, and cytokine-suppressing effects that we attribute to memantine in Section 4.1, Section 4.2, Section 4.3 and Section 4.4, chaperone induction is a plausible—and possibly quantitatively dominant—downstream mediator of memantine’s protective actions in both the central and peripheral nervous system, that has not been directly interrogated in any of the SH-SY5Y, DRG, Schwann-cell, or peripheral-nerve models cited here. Systematic assessment of HSP70 (and of the HSF1 pathway more broadly) in peripheral neural tissue exposed to memantine is therefore a priority for future mechanistic work.

### 10.5. Absence of Transcriptomic Profiling of Memantine-Treated Peripheral Cells

A closely related limitation is the absence of transcriptomic profiling in the studies on which this review draws. Neither the SH-SY5Y oxaliplatin–memantine dataset nor any of the peripheral-nerve neuropathy models discussed in Section 4, Section 5, Section 6, Section 7 and Section 8 have been accompanied by bulk RNA-sequencing, single-cell RNA-sequencing, or spatial transcriptomic analysis of memantine-treated cells or tissues. Because unbiased transcriptomic readouts can directly reveal the molecular targets and effector pathways of a drug—including chaperone systems (HSPA1A/HSPA5/HSPA9, HSF1 targets), Nrf2/HO-1 antioxidant genes, BDNF/TrkB axis components, NF-κB-driven cytokine networks, and NMDA receptor subunit remodeling in DRG and Schwann-cell populations—the lack of such data means that the mechanistic model advanced in this review necessarily relies on candidate-pathway assays selected a priori. Future work should incorporate transcriptomic (and ideally proteomic and phospho-proteomic) profiling of peripheral neural tissue, DRG, satellite glial cells, and Schwann cells at multiple timepoints following memantine exposure in physiological and neuropathic conditions; this would be the most efficient route to identify downstream molecular targets and to directly test the HSP70 hypothesis outlined in Section 10.4.

## 11. Future Directions

### 11.1. Single-Cell Transcriptomics and High-Resolution Receptor Mapping

Advances in single-cell RNA sequencing (scRNA-seq) and spatial transcriptomics now enable comprehensive, cell-type-resolved mapping of NMDA receptor subunit expression across DRG neuron subtypes, Schwann cells, satellite glial cells, autonomic ganglia neurons, and enteric neurons under physiological and neuropathic conditions. Applying these technologies to peripheral nerve and ganglionic tissue from human donors with DPN, CIPN, and other peripheral neuropathies in comparison with healthy controls would establish the disease-specific NMDA receptor subunit landscape that determines the mechanistic basis, and likely the pharmacological response, to memantine [21]. Protein-level validation using super-resolution immunofluorescence microscopy would complement transcriptomic data and enable quantification of receptor surface density, clustering, and subunit stoichiometry at peripheral nerve terminals.

### 11.2. Peripherally Restricted Formulations and Targeted Drug Delivery

The development of peripherally restricted memantine formulations represents a major pharmacological opportunity for improving the therapeutic index of peripheral NMDA receptor blockade. Lipid nanoparticle (LNP) and polymeric nanocarrier systems capable of targeted delivery to peripheral neural tissue following systemic administration could achieve pharmacologically effective local concentrations at DRG and peripheral nerve sites whilst minimizing CNS exposure and associated cognitive and sedative adverse effects [46]. Perineural injectable depot formulations already employed clinically for local anesthetic delivery could be adapted for sustained memantine release at specific nerve injury sites in CIPN or post-amputation neuroma contexts. Transdermal formulations exploiting memantine’s lipophilicity for percutaneous absorption to peripheral cutaneous nerves offer an additional delivery strategy for small-fiber neuropathy applications.

An alternative pharmacological strategy is the development of NR2B-selective NMDA receptor antagonists with limited blood–brain barrier penetration, which would exploit the NR2B-dominant subunit composition of peripheral sensory neurons to achieve peripheral-selective neuroprotection without CNS psychotomimetic effects [19,21]. Compounds in this pharmacological class would provide the definitive tool to validate PNS-specific NMDA receptor-mediated neuroprotection in clinical trials and, if effective, represent a next-generation advance beyond memantine for peripheral neuropathic conditions.

### 11.3. Biomarker Strategies and Personalized Trial Design

Future clinical trials should incorporate PNS-specific structural and functional biomarkers as primary or co-primary endpoints to capture peripheral neuroprotective effects that are invisible to pain score instruments. Established and emerging candidates include the following: intraepidermal nerve fiber density (IENF density) by skin punch biopsy, which is sensitive to small-fiber loss in CIPN and DPN; corneal confocal microscopy (CCM) for non-invasive quantification of corneal nerve fiber length and density as a surrogate of systemic small-fiber neuropathy; nerve conduction velocity and amplitude for large-fiber function; serum and plasma neurofilament light chain (NfL) as a circulating marker of peripheral and central axonal injury with demonstrated sensitivity to treatment effects in multiple neuropathy types; and quantitative sensory testing (QST) thermal and mechanical detection thresholds that specifically quantify small-fiber function [29,47].

Patient stratification based on neuropathy mechanism, NR2B subunit expression profiles (where accessible from skin biopsy material), and serum inflammatory biomarker status would enable mechanistic enrichment of trial populations most likely to respond to peripheral NMDA receptor blockade. The consistent clinical evidence that pre-emptive memantine therapy is more effective than reactive treatment argues for trial designs that enroll patients at the initiation of neurotoxic chemotherapy, the onset of diabetes with early subclinical DPN, or the perioperative period before major amputative surgery, intercepting the injury-sensitization cascade before it consolidates into chronic disease [9,16,42].

### 11.4. Omics-Based Pathway Discovery and Combination Therapeutic Strategies

Transcriptomic and proteomic profiling of peripheral nerve tissue, DRGs, and circulating immune cells following memantine treatment in neuropathy models would identify downstream signaling adaptations, including modifications of BDNF-TrkB axis activity, Nrf2/HO-1 antioxidant pathway induction, mitochondrial biogenesis gene expression, and NF-κB-driven neuroinflammatory mediator profiles, that may predict therapeutic benefit and reveal pharmacological nodes amenable to synergistic co-targeting [28,29]. Combination strategies pairing memantine with gabapentinoid agents (as employed in the DPN RCT), with BDNF supplementation or TrkB agonists, with Nrf2 activators, or with sigma-1 receptor agonists merit evaluation in preclinical peripheral neuropathy models, building on the mechanistic convergence between these agents’ downstream protective effects.

## 12. Conclusions

The evidence reviewed and synthesized in this work makes a compelling and multi-dimensional case for reconceptualizing memantine as a system-wide neuroprotective agent rather than a CNS-restricted pharmaceutical. Across the principal clinically relevant peripheral neuropathy types—diabetic, chemotherapy-induced, traumatic, and post-amputation, the pharmacodynamic logic of voltage-dependent NMDA receptor blockade operates with mechanistic coherence in peripheral sensory neurons, Schwann cells, retinal ganglion cells, and autonomic ganglia. The convergence of preclinical mechanistic data (calcium attenuation, mitochondrial stabilization, NOX-2 suppression, anti-apoptotic signaling) with clinical trial signals across DPN, phantom limb pain, and pre-emptive surgical pain prevention constitutes a substantive evidence base for peripheral memantine neuroprotection that deserves greater clinical and research attention than it has historically received.

The pattern of positive clinical evidence strongest in prophylactic and pre-emptive applications and weaker in established chronic pain states is not evidence of limited efficacy but rather of a pharmacologically rational timing dependency: NMDA receptor blockade prevents the consolidation of central sensitization most effectively when administered before or at the inception of the injury-sensitization cascade. This principle, now supported by mechanistic and clinical convergence, has direct implications for therapeutic strategies in CIPN prevention, DPN management at early stages of peripheral nerve dysfunction, and perioperative analgesia in amputation surgery.

The tolerability profile that distinguishes memantine from other NMDA antagonists and its low propensity for psychotomimetic, dissociative, or cardiovascular adverse effects uniquely suited for prophylactic use in patient populations undergoing prolonged exposure to peripheral neurotoxic stimuli. As peripheral NMDA receptor biology becomes better characterized through single-cell transcriptomic and proteomic approaches, and as peripherally targeted formulation strategies reduce off-target CNS effects whilst improving peripheral tissue concentrations, the full therapeutic potential of memantine for peripheral neuropathy, neuropathic pain, and peripheral organ protection will become increasingly realistic. The conceptual transformation proposed here from memantine as a CNS cognitive agent to memantine as a system-wide neuroprotective compound should motivate a new generation of rigorous, mechanistically informed clinical trials designed to harness its peripheral actions for the benefit of the many patients whose unmet therapeutic needs remain unaddressed by currently available peripheral neuropathy treatments.

## Figures and Tables

**Table 1:** Summary of clinical trials evaluating memantine in peripheral nervous system conditions.

Ref.	Timing	Primary Outcome and Result	Regimen	*n*	Condition	Study
[32]	Prophylactic	↓ DN4 score at 1 and 3 months (*p* = 0.033; *p* < 0.01); 40% vs. 10% neuropathy-free	Memantine 20 mg/day × 8 wks vs. no memantine	40	CIPN (docetaxel, breast cancer)	Mohammadzadeh 2025
[9]	Active treatment	↓ DN4, ↑ vibration/monofilament/thermal thresholds (all *p* < 0.001)	Memantine 10 mg BID + gabapentin 300 mg/day vs. gabapentin monotherapy × 8 wks	143	DPN (T2DM)	Jafarzadeh 2023
[16]	Peri-amputation	↓ PLP intensity at 4 wks (VAS 3.4 vs. 24) and 6 mos (VAS 7 vs. 17); NS at 12 mos	Memantine 10 → 30 mg/day × 4 wks vs. placebo	36 (18/arm)	Chronic phantom limb pain	Maier 2003
[34]	Perioperative	Memantine only agent with significant pooled effect at 6 mos (MD −0.70, 95% CI −1.23 to −0.18)	Systematic review of perioperative pharmacological interventions	Pooled	Perioperative PLP prevention	Abrishami 2025 (meta-analysis)
[35]	Active treatment	↓ Pain with movement, mood, and disability; ↓ pain-evoked S1/S2 fMRI activation	Memantine 5–40 mg/day × 49 d + morphine + physiotherapy vs. morphine + physiotherapy	20 (10/arm)	CRPS type I/II	Gustin 2010
[44]	Prophylactic	↓ Post-mastectomy pain intensity at 3 mos (*p* = 0.017); 5% vs. 30% needed neuropathic pain treatment (*p* = 0.04)	Memantine 5 → 20 mg/day × 4 wks (2 wks pre-, 2 wks post-op) vs. placebo	40 (20/arm)	Post-mastectomy neuropathic pain	Morel 2016
[42]	Established pain	No significant benefit versus placebo	Memantine 10–20 mg/day, 5 wks	24; various	Established PHN and painful DPN	Eisenberg 1998
[21]	Maintenance	No sustained analgesic difference at 3 mos; memantine improved cognition and QoL sub-scores	Dextromethorphan 90 mg/day, memantine 20 mg/day or placebo × 12 wks	60	Refractory neuropathic pain post-IV ketamine	Martin 2019

↓: reduction, ↑: increase. Abbreviations: CIPN, chemotherapy-induced peripheral neuropathy; CRPS, complex regional pain syndrome; DN4, Douleur Neuropathique 4 questionnaire; DPN, diabetic peripheral neuropathy; MD, mean difference; PHN, post-herpetic neuralgia; PLP, phantom limb pain; T2DM, type 2 diabetes mellitus; VAS, visual analog scale; fMRI, functional magnetic resonance imaging; QoL, quality of life.

## Data Availability

No new data were created or analyzed in this study. Data sharing is not applicable to this article.
